# Field Evaluation of Advanced Rice Lines for Adaptability to Drought and Heat in the Senegal River Valley

**DOI:** 10.1002/pei3.70034

**Published:** 2025-02-16

**Authors:** Yonnelle Dea Moukoumbi, Raafat El‐Namaky, Mouritala Sikirou, Roland Bocco, Daouda Mbodj, Esther Pegalepo, Adoté Hervé Gildas Akueson, Baboucarr Manneh

**Affiliations:** ^1^ Institut de Recherches Agronomiques et Forestières (IRAF) Libreville Gabon; ^2^ Irrigated Rice Breeding Unit Africa Rice Center (AfricaRice), Sahel Regional Station Saint‐Louis Senegal; ^3^ International Institute of Tropical Agriculture Kinshasa Democratic Republic of the Congo; ^4^ Texas AgriLife Research and Extension Center Texas A&M Univ., System Beaumont Texas USA; ^5^ Africa Rice Center (AfricaRice), Mbe Station Bouake Côte d'Ivoire; ^6^ Unité de Statistique et d'Informatique Appliquées USIA/Laboratoire d'études et de Recherche Forestière (LERF) Faculté d'Agronomie Université de Parakou (FA‐UP) Parakou Benin

**Keywords:** extreme temperature, rice advanced lines, water scarcity, yield response

## Abstract

In Senegal, the average rice consumed is 100 kg per capita per year. The objective was to evaluate and select the well‐adapted high‐yielding lines in Ndiaye and Fanaye growth conditions in Senegal River Valley. One hundred and twelve advanced lines were evaluated in consecutive wet and dry seasons at AfricaRice Fanaye and Ndiaye sites challenged by drought and high temperatures. Unlike Fanaye, Ndiaye faces severe water scarcity and extreme heat. An alpha‐lattice design was used with three replications. The number of tillers and plant height at 30 days after sowing, plant height at maturity, days to 50% heading, and grain yield; physiological: leaves chlorophyll content at 50% heading stage, yield grain, thousand grain weight, and number of panicles per plant were recorded to evaluate the increasing of rice productivity. Results showed significant variation among the advanced lines and the test “Kruskal‐Wallis medians” was used for the mean comparison for the five descriptors during growth and development stages. Path analysis revealed that Ndiaye's harsh conditions negatively impacted NT30, PH30, PHmat, PNP, Dmat, and GY, with negative effects on NT30 (*ρ* = −0.63), PH30 (*ρ* = −0.67), and PNP (*ρ* = −0.15). However, SH (*ρ* = 0.71) and TGW (*ρ* = 0.37) had positive direct effects. Cluster analysis generated four groups showing the characteristics of 112 advanced lines. Most of the advanced lines were outperforming local elite varieties. The lines WAC 18‐WAT15‐3‐1, WAC 18‐WAT65‐1‐1, WAC 13‐WAT32‐2‐1, and WAB 2150‐TGR1‐WAT3‐1 produced the highest yields for Ndiaye, with 4752, 5589, 5589, 5644, and 6943 kg/ha. For Fanaye, the best genotypes were IR 09 N523, CT18919‐4‐2‐2‐2SR‐1P, CT18494‐4‐4‐3‐3‐1SR, WAB 2125‐WACB‐1‐TGR1‐WAT1‐1, and CT19541‐13‐3‐1‐2P‐3P, with 8824, 8984, 9014, 9639, and 8496 kg/ha, respectively. The authors recommend that these lines be released or used as donors in breeding programs, and further studies can consider stability analysis using the best adapted varieties.

## Graphical abstract

Advanced lines grain yield response drought and heat effects evaluation.

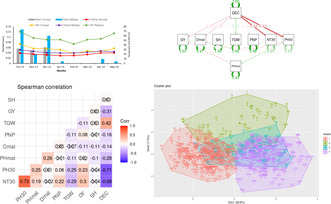

## 1 Introduction

Rice is the world's most important crop, accounting for more than 50% of total human intake (Fukagawa and Ziska 2019; FAO 2019). In West Africa, this crop is an essential food security commodity (Bandumula 2018; Blein Soule et al. 2008). Rice consumption in this region has surged as a consequence of population increase, urbanization, and higher demand (Zhou and Staatz 2016; Africa Rice Center 2007). The sub‐region's average consumption per capita increased from 32 kg in 1990 to 85 to 100 kg in 2024 (Glatzel 2018). However, the region continues to have a rice shortage, with domestic production accounting for barely 60% of overall consumption. Senegal, Ghana, Benin, and Côte d'Ivoire display less than 40% self‐reliance in rice (Fofana et al. 2014). Nigeria, Senegal, Côte d'Ivoire, and Benin provide over 50% of West Africa's rice imports. Despite the continent's abundant fertile land and agricultural potential, the ability to meet expanding rice demand is becoming increasingly limited. As a result, there is a greater reliance on imports to bridge the production‐consumption imbalance. Africa continues to import up to 12.6 million metric tons of milled rice per year, representing around US$5.5 billion (Adekunle 2023). According to Soullier et al. (2020), West African countries including Senegal, Guinea‐Bissau, and Guinea depend heavily on rice as a source of energy.

Crop intensification, or boosting productivity on current cropland, might help lessen the need for expensive food imports and/or alleviate pressure on converting land to agriculture (Van Oort et al. 2015; Van Ittersum et al. 2016; Silva et al. 2022). Unfortunately, this crop must deal with biotic stresses like insects (IRRI et al. 2010; Zenna et al. 2017; Bocco et al. 2012), granivorous birds (De Mey et al. 2012), mice (Stenseth et al. 2003), parasitical nematodes (Khan et al. 2023), and weeds (Moukoumbi et al. 2011; Rodenburg et al. 2016). Rice is also subject to abiotic stresses: water deficiency (Bocco et al. 2012; Adjah et al. 2022), floods (Mwakyusa et al. 2023), iron toxicity (Audebert and Fofana 2009), salinity (Van Oort 2018), and extreme temperature (De Vos et al. 2023). In West Africa, climate change is likely to increase in frequency and amplitude, whereas the annual rainfall expected is declined and increased (Diedhiou et al. 2018). These aforementioned factors affect rice cultivation and yield in Africa. FAO (2022) reports that Africa's average rice production stands at 2.4 t/ha, a 33% decrease from Southeast Asian farmers' output of 4.3 t/ha.

Climate change poses growing hazards to the Senegal River Valley, including rising temperatures, irregular rainfall, droughts, floods, sea level rise, soil and water salinization, desertification, and land degradation (Faye et al. 2021; Agulonye 2023; Madurga‐Lopez et al. 2022). Mali, Mauritania, and Senegal all share boundaries with this region (Faye 2023; Taïbi et al. 2023).

Researchers and African governments are taking steps to develop or introduce resilient varieties to assist the government to implement political measures to attain rice self‐sufficiency (Brosseau et al. 2021). Senegal's government, for example, has established public investments, price regulation, and expanded outreach activities (Voss 2020). By 2035, the national long‐term economic growth policy seeks to have the country's rice output at maximum (Cisse 2024). The efforts are focused on irrigated and rainfed rice, and rainfed rice agriculture in Senegal has grown considerably, particularly in the Kaffrine, Senegal Oriental, and Casamance regions, following a significant reduction during the century's dry decades (Gerardeaux et al. 2021; Cisse 2024). Despite significant government commitments to enhance rice production, water, soil, and crop management constraints continue to limit paddy yields (Faye et al. 2021). Furthermore, researchers have developed high‐yielding lines with desirable traits to reduce losses in yield by limiting rice sterility at high temperatures (Jagadish et al. 2015). These traits include better transpiration cooling, heading early in the morning, phenological shifts that lower stress, more spikelets, and stronger sinks (Basavaraj and Rane 2020; Senguttuvel et al. 2022; Sheehy et al. 2001). Even so, research is ongoing to create novel varieties that may help African countries achieve food self‐sufficiency after determining the plant's genetic diversity (Shilomboleni and Ismail 2023).

The study aimed to: (i) examine the grain yield and yield components related traits of advanced lines grown under irrigated conditions in the Senegal River Valley; (ii) investigate the impact of different traits on yield improvement; and (iii) identify the performing high yielding advanced lines that could serve as donors in breeding programs. Several statistical methods were carried out to determine yield response, genotypic variation, effects, and relationships between the examined parameters. Few research studies have been conducted to evaluate many advanced lines to counteract the effects of climate change in both Ndiaye and Fanaye AfricaRice experimental stations.

## 2 Materials and Methods

### 2.1 Plant Material and Experimental Establishments

The experiments were conducted during the 2013 wet season and 2014 dry season and under irrigation conditions at Africa Rice Sahel stations in Ndiaye (16°14′N, 16°14′W, and 9 m altitude) and Fanaye (16°31′59′N, 15°13′59′W and 30 m altitude). The delta inland of the Senegal River Valley, where the two research sites are located, is susceptible to flooding, drought, and warming due to climate change. One hundred and twenty rice advanced high‐yielding lines collected with National Agricultural Research System (Senegal, Mali, and Burkina Faso), Africa Rice Center (AfricaRice), Biodiversity Center, Breeding Taskforce, Empresa Brasileira de Pesquisa Agropecuaria (EMBRAPA), and International Network for Genetic Evaluation of Rice at International Rice Research Institute (INGER‐IRRI) were used, with eight local elite varieties: DKA 21, DKA 22, Morobekan, IR 68, IRRI 123, IR 50, Sahel 108, and NERICA‐S‐36 serving as controls. Those varieties were chosen for their high production, good taste, and resilience to stress. The 112 advanced lines were previously evaluated throughout the MET (Multi Environmental Trials) under Breeding Taskforce countries to determine their agronomic performance and tolerant to abiotic and biotic stresses related to rice production. The Africa Rice Center Breeding Taskforce approach is a rigorous procedure that considerably reduces the field selection time of advanced lines, which can be summarized as follows: all the lines are tested in the field for one season, using a procedure commonly known as Observational Yield Trials (OYT). At this stage, the breeder monitored all the lines to get an overall opinion. The Preliminary Yield Trial (PYT) uses an Randomized Complete Block Design (RCBD)** experimental design, and the breeder collected agromorphological data for each plotusing the plant selected. Upon completion of this evaluation, the breeder harvested the lines demonstrating excellent yield potential to advance them to Advanced Yield Trial (AYT). Here, the plots are large, and the number of samples used for each advanced line is also important. Typically, during this stage of the study, the breeder retains around 20% of the evaluated population. After AYT, the advanced lines selected are shared through Breeding Taskforce of the member countries where each institution uses the lines adapted to its agro‐environmental conditions for a probable future approval through AfricaRice Breeding Taskforce (shuttle breeding).

Countries have subsequently adopted and continue using several of the advanced lines evaluated during this study.

Table S1 listed in the annex presents all the plant materials tested during two seasons in two locations. The experiments were carried out using an alpha lattice design and three replications of six blocks, each containing 20 advanced lines including the checks varieties. The transplanting was conducted 21 days after sowing (DAS) in a 1 × 2 m plot with 10 rows. Each hill contained a single plant with a 20 × 20 cm space within and between rows.

### 2.2 Field Management

Irrigation was utilized permanently after crop establishment until at least two weeks after flowering (vegetative and reproductive stages included). The water level in the plot was constant 5–10 cm. We recorded daily temperature and relative humidity patterns from transplantation to the evaluation end using an Onset HOBO (U12 Temp/RH/2 External Channel Logger, Onset Computer Corporation, 470 MacArthur Boulevard, Bourne). During the assessments, we collected and measured the amount of liquid precipitation across the two sites using a rain gauge (Holman Industries NZ RGC1000 Professional Rain Gauge). For Ndiaye, some arrangements were performed to prevent salinity content in the plots. One week before transplanting, the plots were flooded and salinity content was taken for Ndiaye (once per week, and the plots were immediately drained when the salinity content was greater than 1).

During the land preparation, the vegetative and reproductive stages, fertilizers (NPK_15‐15‐15_ and Urea) were applied following AfricaRice recommendations. The best agronomic practices were used to mitigate damage caused by insect, pest, disease, and bird attacks.

### 2.3 Data Collection

Agromorphological and yield component traits were recorded using rice descriptors (IRRI‐AfricaRice‐Biodiversity 2013). Plant height at 30 days after sowing (PH30) was measured from topsoil to panicles. The number of tillers per plant at 30 DAS (NT30) was manually counted and recorded. Panicle numbers per plant (PNP) were calculated by recording panicles on ten randomly selected plants and taking the average. Thousand grain weight (TGW) was measured after harvest. The total chlorophyll leaf content after heading (SH) was measured at 50% heading stage using a SPADmetter (SPAD‐502Plus by Konica Minolta Inc., in the United States). Days to maturity (DM) were counted from the day of sowing until 95% panicle maturity. Harvesting 10 plants from the same region identified per elementary plot to calculate grain yield weight (GY) (kg) at 14% moisture content.

### 2.4 Statistical Analysis

Data were analyzed using the free and open‐source software R 4.3.2. Descriptive Statistics and the Kruskal–Wallis median analysis test were performed. We studied correlations of variables such as the NT30, PH30, PHmat, SH, Dmat, PNP, TGW, and GY were generated with the “stats” package. The “ggcorrplot” software was used to represent the correlogram. The “FactomineR” and “factoextra” packages were used to perform and develop the hierarchical classification model on the quantitative traits. Path analysis allowed us to assess direct and indirect relationships between a set of variables of interest. This analysis required the use of the “lavaan”, “semPlot”, “OpenMx”, and “GGally” packages.

## 3 Results

### 3.1 Evolution of Weather Patterns

**FIGURE 1:**
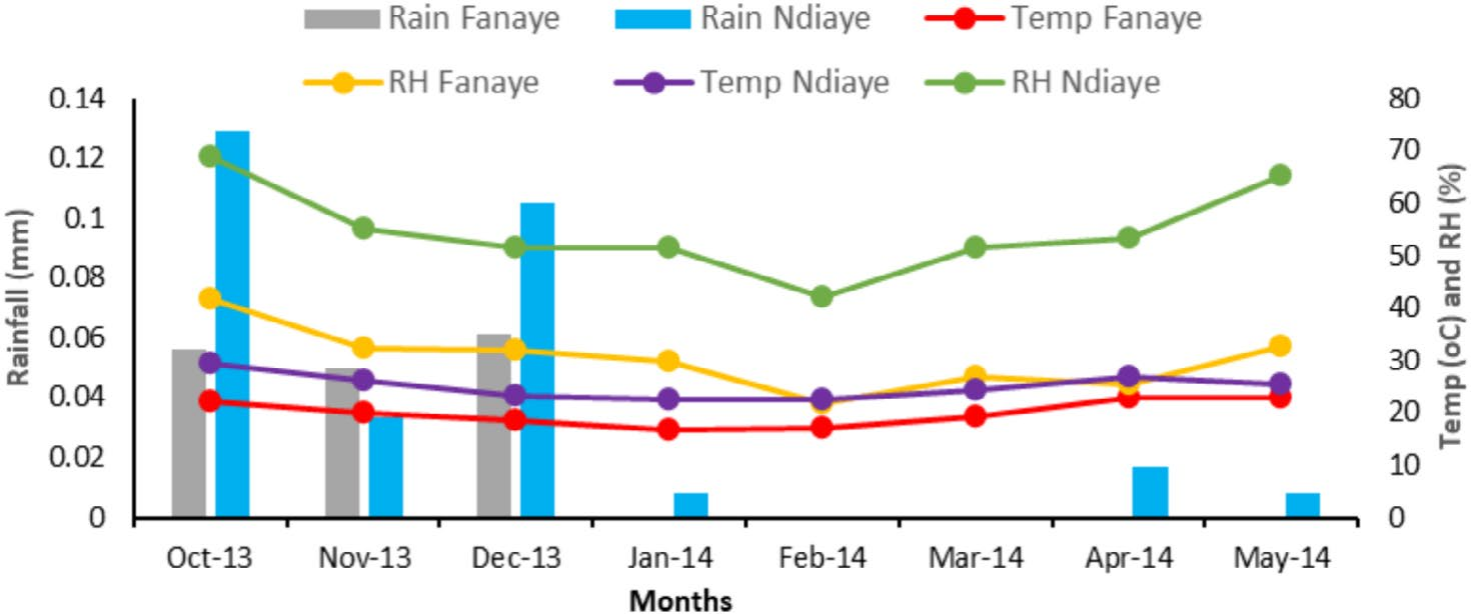
The weather during the experiments in Fanaye and Ndiaye. Rain Fanaye: Rainfall Fanaye; Temp Ndiaye: Temperature Ndiaye; RH Fanaye: Relative humidity Fanaye. Rain Ndiaye: Rainfall Ndiaye; Temp Fanaye: Temperature Fanaye; RH Ndiaye: Relative humidity Ndiaye.

Figure 1 shows the weather data collected during the field studies at Ndiaye and Fanaye. The average temperature trend was the same between the two experimental sites. However, plants were grown under higher (*p* = < 0.05) relative humidity in Ndiaye, contrary to Fanaye. Both locations had some precipitations, but Ndiaye had less.

### 3.2 Data Analysis

Shapiro–Wilk normality tests performed at the 5% threshold on the advanced lines and eight checks varieties' growth characteristics provide *p*‐values equal to 0.001. As a result, the quantitative variables' normal assumptions are 99.99% confidently rejected. In this scenario, the Kruskal‐Wallis test is a substitute for the analysis of variance.

There was a major difference between the genotypes in the NT30, PH30, PHmat, SH, Dmat, PNP, TGW, and GY. Table 1 presents the Kruskal–Wallis tests on the median values of the genotypes' growth characteristics.

**TABLE 1:** The significance test based on a comparison of the Kruskal–Wallis medians.

Characters	Abbreviation	Mean sq	df	Probability level, Pr (> *F*)
Tiller numbers at 30 DAS (number)	NT30	330.5	1	0.001***
Plant height at 30 DAS (cm)	PH30	361.7	1	0.001***
Plant height at maturity (cm)	PHmat	54.7	1	0.001***
SPAD at 50% heading (SPAD unit)	SH	372.4	1	0.001***
Days to maturity (days)	Dmat	14	1	0.001***
Panicle per plant (number)	PNP	18.1	1	0.001***
Thousand grain weight (g)	TGW	129.3	1	0.001***
Grain yield (kg/ha)	GY	68.1	1	0.001***

*** High significance (*p* < 0.0001).

### 3.3 Evaluation of Phenotypic Diversity of 112 Advanced Lines and Control Varieties

The examination of these variables' distributions revealed the phenotypic diversity observed in the prior quantitative characters analyzed. Table 2 displays the results of the numerous detected patterns.

**TABLE 2:** Phenotypic diversity performed using agro‐morphological traits.

Characters	Fanaye (*N* = 360)	Ndiaye (*N* = 360)	Rate of change of characteristics in Ndiaye compared with Fanaye	Overall (*N* = 720)
NT30				
Mean (SD)	7.49 (3.16)	3.44 (1.60)	−54.07%	5.47 (3.22)
Median [min, max]	7.00 [1.25, 17.5]	3.25 [1.00, 9.75]	−53.57%	4.75 [1.00, 17.5]
PH30				
Mean (SD)	35.0 (4.72)	26.0 (5.30)	−25.71%	30.5 (6.75)
Median [min, max]	35.3 [0, 49.8]	26.3 [10.3, 43.3]	−25.50%	31.0 [0, 49.8]
PHmat				
Mean (SD)	117 (17.7)	107 (14.0)	−8.55%	112 (16.7)
Median [min, max]	114 [79.5, 198]	107 [73.0, 150]	−6.14%	111 [73.0, 198]
SH				
Mean (SD)	22.4 (7.45)	34.9 (4.77)	55.80%	28.6 (8.85)
Median [min, max]	22.0 [4.73, 44.8]	34.8 [22.1, 44.8]	58.18%	29.7 [4.73, 44.8]
Dmat				
Mean (SD)	142 (10.6)	140 (8.84)	−1.41%	141 (9.81)
Median [Min, Max]	145 [105, 155]	143 [103, 163]	−1.38%	143 [103, 163]
PNP				
Mean (SD)	20.3 (6.78)	18.5 (5.05)	−8.87%	19.4 (6.04)
Median [min, max]	19.3 [4.75, 61.8]	17.9 [5.50, 39.3]	−7.25%	18.8 [4.75, 61.8]
TGW				
Mean (SD)	23.5 (3.05)	26.5 (4.49)	12.77%	25.0 (4.13)
Median [min, max]	23.3 [2.16, 31.9]	26.6 [2.49, 32.8]	14.16%	24.8 [2.16, 32.8]
GY				
Mean (SD)	8410 (1710)	7340 (2100)	−12.72%	7870 (1990)
Median [min, max]	8940 [187, 9990]	7860 [642, 10,300]	−12.08%	8510 [187, 10,300]

Abbreviations: Dmat, days to maturity; GY, grain yield; NT30, tiller numbers at 30 DAS; PH30, plant height at 30 DAS; PHmat, plant height at maturity; PNP, panicle number per plant; SH, SPAD at 50% heading; TGW, thousand grain weight.

At the Fanaye, the number of tillers per plant and plant height at 30 days after sowing (DAS) ranged from 1.25 tillers to 17.5 tillers and 0 to 49.8 cm, with averages of 7.49 ± 3.16 tillers and 35 ± 4.72 cm for plant height. The Ndiaye site produced fewer results, with tillers ranging from 1.00 to 9.75 and heights ranging from 10.3 to 43.3 cm per plant, with an average of 3.44 ± 1.60 tillers and 26.0 ± 5.30 cm per plant. After comparing the two sites, it was discovered that the number of tillers and plant heights at 30 DAS in Ndiaye were −54.07% and −25.71% lower than in Fanaye, respectively.

Rice plants matured at 142 ± 10.6 DAS in Fanaye and 140 ± 8.84 DAS in Ndiaye. Throughout the maturity period, Fanaye had higher average plant heights (117 ± 17.7 cm) than Ndiaye (107 ± 14.0 cm). Fanaye had a chlorophyll content of 22.4 ± 7.45, while Ndiaye had 34.9 ± 4.77. This scenario corresponds to a 55.80% increase in chlorophyll content for Ndiaye. Fanaye showed a substantially higher average number of panicles per plant. Investigations in the Ndiaye locality yielded roughly 18.5 ± 5.05 seeds, compared to 20.3 ± 6.78.

The average grain yield (GY) in Fanaye was 8410 ± 1710 kg/ha, whereas in Ndiaye it was 7340 ± 2100 kg/ha. As a result, the Ndiaye site produced 12.72% less than the Fanaye site. However, weight analysis of the seeds showed a 12.77% increase on the Ndiaye site compared to Fanaye. The average grain weight was 58.8 ± 20.4 kg for the Fanaye site and 26.5 ± 4.49 kg for the Ndiaye location.

### 3.4 Correlation Coefficients

**FIGURE 2:**
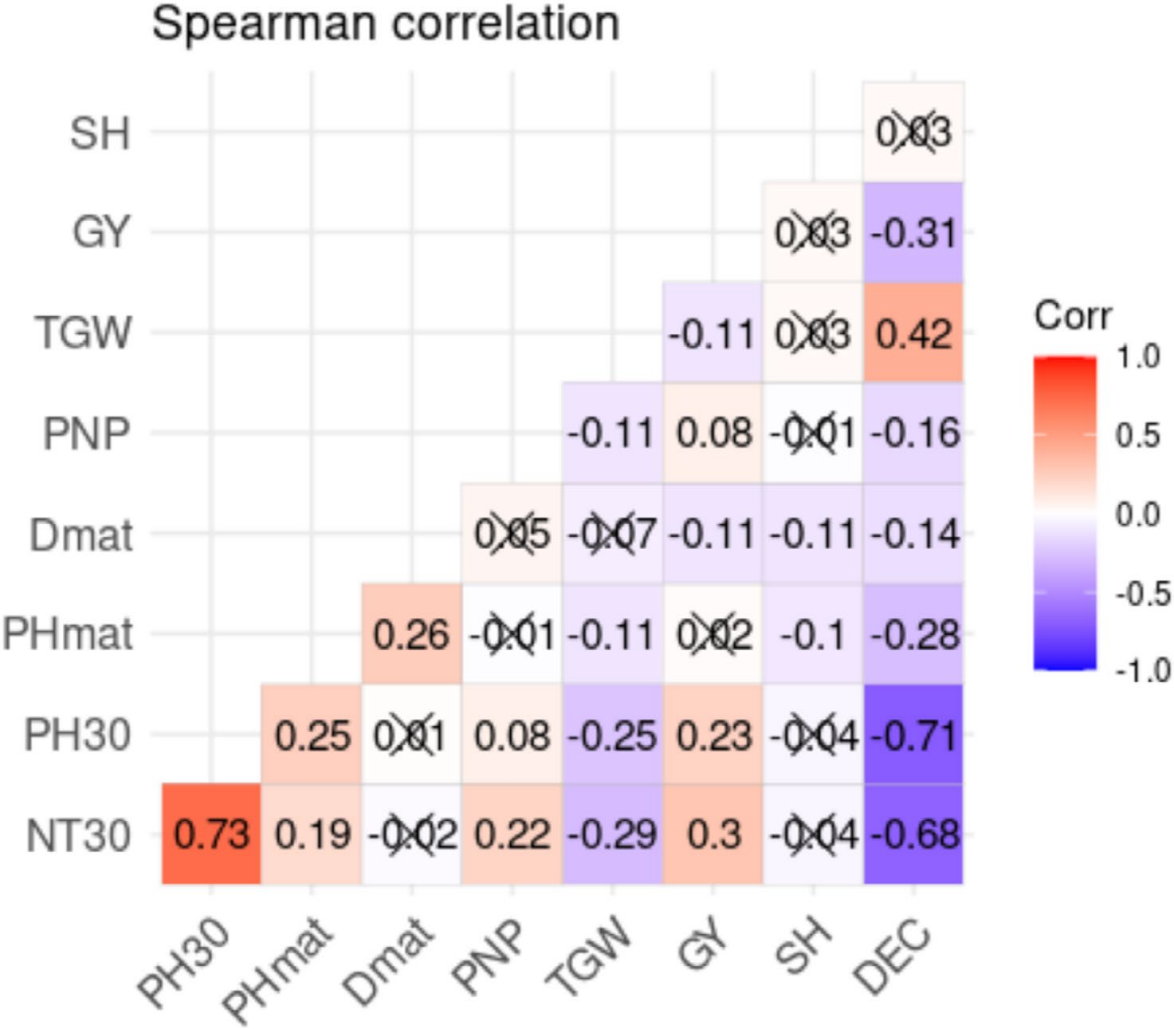
Correlogram based on different color variations from −1 to 1 at a significance level between the eight characters (NT30, PH30, PHmat, Dmat, GY, SH, PNP, and TWG), and difference in cultivation environment (DEC).

The Spearman correlation coefficients (*n*) of 8 quantitative traits (Figure 2) indicated a negative impact on the number of tillers at 30 DAS (NT30), the height of the plants at 30 DAS (PH30), the plant's height at maturity (PHmat), the number of panicles per plant (PNP), the date of maturity (Dmat), and the grain yields (GY) when moving from Fanaye to Ndiaye. Rice production in the Ndiaye region, as opposed to Fanaye, is associated with higher chlorophyll content after heading (SH) and thousand grain weight.

Rice farming in Ndiaye negatively correlated with the number of tillers, the plant height, the number of panicles per plant, and the grain yield (kg/ha). However, the Ndiaye site possessed characteristics that encouraged an increase in leaf chlorophyll content and thousand grain weight. The investigation of the intensities of the correlations allows to notice that the particularities observed in the Ndiaye region have a considerable negative effect on the number of tillers (*ρ* = −0.68) and plant height (*ρ* = −0.71) at 30 days after planting. Furthermore, its effect steadily decreased throughout the plant's growth phase. However, the Ndiaye environment had a positive and substantial effect on leaf chlorophyll content (*ρ* = 0.72) and thousand grain weight (*ρ* = 0.42) at maturity.

### 3.5 Path Analysis

**FIGURE 3:**
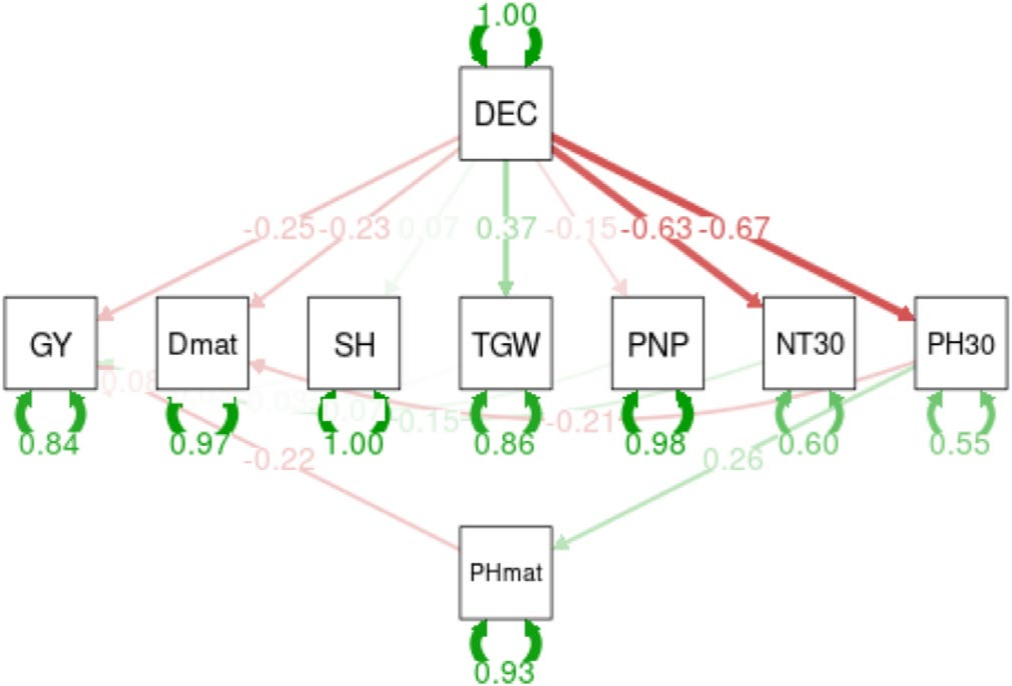
Path diagram between the eight characters (NT30, PH30, PHmat, Dmat, GY, SH, PNP, and TWG), and difference in cultivation environment (DEC).

In Fanaye or Ndiaye, the analysis of the paths reported in Figure 3 for the variables studied exhibited direct and indirect effects on the grain yield of cultivated high‐yielding rice genotypes.

According to this analysis, the transition to an environment like the Ndiaye region displayed a strong direct and negative influence on the number of tillers (−0.630) at 30 days of growth, the number of panicles per plant (−0.15), and the plant height (−0.67) at 30 days of growth. However, the cultivation of high yielding rice accessions in Ndiaye had a direct and favorable impact on chlorophyll concentration (0.71) and thousand grain weight (0.37).

Thus, growing rice in the Ndiaye environment inhibited an increase in the number of tillers and the plant height. This delay in the growth of both the tiller number and the plant height resulted in plants that were 22% shorter in plant height during maturity compared to plants grown in the Fanaye region.

Depending on the growth process, cultivating rice after maturation on the Ndiaye site results in a shortening of the maturity date (−0.23%), a substantial decrease in the plant height (−0.17%), a diminution in the panicle number per plant (−0.15%), and a 26% reduction in yields.

### 3.6 Clusters Analysis

**FIGURE 4:**
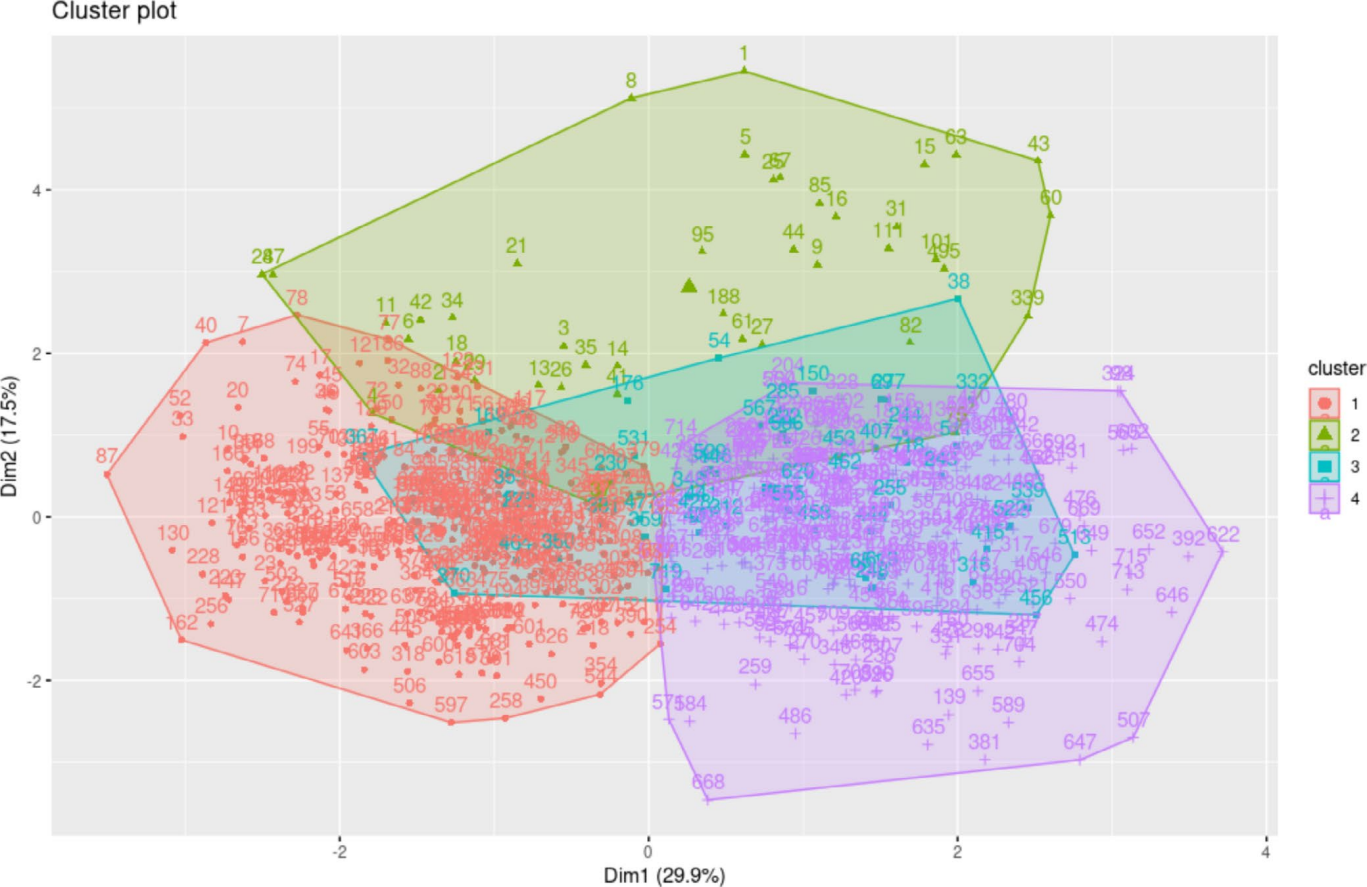
Cluster analysis and characterization of genotype groups.

Cluster analysis classified the genotypes into four homogeneous groups (Figure 4). Plants grown in Ndiaye were split into groups 1 and 3, whereas those developed in Fanaye were divided into groups 2 and 4.

The first category was made up of 46.94% of the population evaluated. It contains 94.38% of the rice genotypes cultivated in Ndiaye. Group 1 consisted of genotypes exposed to the Ndiaye environment, resulting in a significant yield loss of 10.91%. This set can be classified as a collection of less efficient genotypes in the Ndiaye environment.

The second group accounted for 5.83% of the genotypes in the study. It was distinguished by 66.67% of genotypes cultivated at the Fanaye location. This category included genotypes with a priori low grain yield.

The third group, which comprised 7.5% of the population analyzed, was made up of 70.37% of the rice genotypes cultivated in Ndiaye. Plants in this group, subjected to Ndiaye's growing conditions, experienced a lower yield loss than those in the first classification group. This set can be classified as a group of genotypes that were more suited to the Ndiaye environment.

The fourth category included 39.72% of the genotypes in the study. They represented 96.15% of the genotypes planted at the Fanaye location, which distinguished the category. This category consisted of genotypes that had a larger grain yield than the third group. This collection can be defined as the most efficient genotypes on the Fanaye site.

Table 3 reported the agromorphological characteristics of the four groups generated by cluster analysis.

**TABLE 3:** Agromorphological characteristics of the four groups.

Characters	Group 1	Group 2	Group 3	Group 4
NT30 (number)	3.35 ± 1.53	5.15 ± 2.48	5.21 ± 2.30	8.06 ± 3.08
PHmat (cm)	106 ± 13.1	145 ± 26.9	119 ± 12.7	113 ± 12.3
PNP (number)	18 ± 4.47	14 ± 5.17	32 ± 8.92	19.4 ± 3.73
Dmat (days)	140 ± 8.69	146 ± 7.92	149 ± 5.21	140 ± 11.1
GY (kg/ha)	7510 ± 1910	3400 ± 1980	8330 ± 1330	7870 ± 990

Abbreviations: Dmat, days to maturity; GY, grain yield; NT30, tiller numbers at 30 DAS; PHmat, plant height at maturity; PNP, panicle number per plant.

Table 4 shows the genotypes that define each group based on the average of grain yield, harvest index, 1000 grain weight, and distance from the class's barycenter.

**TABLE 4:** Distribution of paragons from each group.

Groups	Genotypes	GY	TGW	Centroid
1	WAC18‐WAT65‐1‐1	5861.34	23.07	1.84
	WAB2099‐WAC2‐1‐TGR2‐WATB3	7894.53	29.11	1.88
	FAROX 521‐357‐H1	6096.19	22.45	1.89
	IR84649‐81‐4‐B‐B	6525	24.06	2.05
	WAB2135‐WAC B‐2‐TGR2‐WAT1‐1	5231.69	27.94	2.06
2	WAB2081‐WAC2‐2‐TGR2‐WAT1‐8	8583.14	28.94	1.47
	CT19558‐2‐17‐4P‐3‐1‐1‐M	7813.14	26.23	1.69
	CT18494‐4‐4‐3‐3‐1SR	6915.70	28.18	1.70
	WAB2102‐WAC3‐1‐TGR1‐WAT B7	9408.37	27	1.73
	WAB1468‐14N‐B‐FKR5‐FKRB	8637.79	26.01	1.80
3	WAC18‐WAT15‐3‐1	5682.64	20.11	2.37
	WAC18‐WAT65‐1‐1	2790.28	20.70	2.57
	WAC13‐WAT32‐2‐1	4423.61	21.54	2.65
	WAB2150‐TGR1‐WAT3‐1	3383.33	22.78	2.86
	WAC18‐WAT65‐1‐1	4501.39	22.85	2.89
4	IR09N523	8687.15	23.30	1.09
	CT18919‐4‐2‐2‐2SR‐1P	8699.31	25.50	1.75
	CT18494‐4‐4‐3‐3‐1SR	9068.75	22.59	1.82
	WAB 2125‐WACB‐1‐TGR1‐WAT1‐1	9936.46	21.70	1.85
	CT19541‐13‐3‐1‐2P‐3P	7806.94	23.53	1.85

Abbreviations: GY, grain yield; TGW, thousand grain weight.

## 4 Discussion

The rice genotypes explored during this field experiment in Ndiaye and Fanaye differ statistically in all parameters studied. This trend in Table 1 indicates the phenotypic diversity of the advanced lines and varieties that comprise the investigated plants. These plant materials are derived from a variety of crossings involving biparental or more parents using Backcross or Marker‐Assisted Recurrent Selection method collected from NARS partners (ISRA, EIR, INERA), “EMPRABA, AfricaRice, IRRI,” one CGIAR center, and breeding Taskforce involving breeders from African countries. As a result, each advanced line will include numerous genes responsible for distinct characteristics inherited from the crossed parents. This study found a difference between the accessions in terms of the total number of tillers at 30 DAS (NT30), plant height at the same date (PH30), and plant maturity (PHmat). This observation demonstrates that during different growth stages, some plants were tall, medium, and short, while others had high, medium, and low tiller numbers. Similarly, the genotypes differed in terms of leaf chlorophyll content at 50% heading stage (SH) and physiological maturity dates (Dmat). Their leaves varied in color from pale green to pure green, with maturity ranging from early to late. The findings showed that after the heading, the plants exhibited a high, medium, and low number of panicles. After harvest, the genotypes varied in grain yield (GY) and thousand grain weight (TGW). In short, all of the plant material analyzed exhibited a high level of variation across all the traits studied. As a result, these genotypes will be extremely valuable in the selection and identification of new plant type of models capable of mitigating the effects of climate change, which affects both locations. Breeders consider the evaluated traits to be components of rice yield. Aside from the considerable diversity between lines for genotypes for the traits studied, the research also showed the impact of the environment on the expression of the characters. According to Table 2, the Fanaye environment was advantageous for the tiller numbers appearing at 30 DAS (NT30), plant height at 30 DAS (PH30), plant height at maturity (PHmat), physiological maturity date (Dmat), number of panicles per plant (PNP), and grain yield (GY). On the other hand, the environment at Ndiaye was favorable for leaf chlorophyll content at 50% heading stage (SH) and thousand gain weight (TGW). This study's findings support those of other researchers who have conducted similar investigations on plant characterization for stress tolerance and improving yields. Plant materials with either early or late maturity can serve various purposes in breeding programs. Early maturity genotypes can avoid stress intervals (Hussain et al. 2022), resulting in drought avoidance in rice. According to Rasheed et al. (2022) and Zeng et al. (2002), an early‐maturing attitude can minimize the adverse effects of salt stress on rice. Li et al. (2018) cited that the interaction of environmental conditions and genotype influences major agronomic traits in rice, including yield, quality, and resistance. However, investigations on environmental factors affecting agronomic features are sometimes difficult to conduct because most environmental elements are dynamic and constantly altering. Huang et al. (2021) conducted several environmental studies and found that drought stress impacted the quantity of panicles per plant and the grain weight. However, Yang et al. (2023) found that crop chlorophyll content can rise or decrease in response to drought stress, with the degree of change generally closely associated with drought resistance. Based on this second assumption, some of the selected genotypes must be drought‐tolerant, which should be beneficial to farmers. Our two research sites are known high temperatures at night, contrary to the rest of the rice cultivation areas (Djaman et al. 2019). Sandhu et al. (2024) and Impa et al. (2021) cited that global nighttime temperatures are a challenge for rice ( *Oryza sativa* ) production and affect rice yield by reducing grain weight, size, and fertility.

At 30 DAS in Ndiaye, the path analysis results revealed that the production environment had a direct negative effect on tiller numbers, panicle numbers, and plant heights. However, the production environment had a negative impact on the leaf chlorophyll content and the thousand grain weight. Plant height and tiller numbers were reduced by 22%, which hampered productivity. Nevertheless, genotypes on this same experimental site matured faster than Fanaye, despite a loss in panicles and yields (Figures 2 and 3). This highlights the impact of environmental and production conditions on long‐term rice cultivation for food self‐sufficiency. It also underscored the necessity of conducting research to create new rice varieties that align with endogenous realities. Despite the fact that Ndiaye and Fanaye are both in the same region with differences related to the values of temperature (over 45°C), they have distinct influences on rice agromorphological characteristics (Table 3). Yang et al. (2022) stated that the quantity of tillers recorded in wheat varied based on rainfall and soil humidity. Janssen et al. (2014), Galvão and Fankhauser (2015), and Liu et al. (2021) mentioned that rice growth and development require specific environmental factors such as light, temperature, water, and soil fertility. According to these authors, biotic and abiotic stresses in the environment have an impact on rice growth and development. Galvão and Fankhauser (2015) and Liu et al. (2021) found that ambient light regulates plant growth through the signals of far‐red light (FR) and red light (R). According to Liu et al. (2021), the R/FR ratio in the natural environment varies with day and night succession, season variation, and crop density. Plants adapt to these changes by modifying their morphology, which can have an impact on yields and yield components. The atmosphere in Ndiaye had been conducive to an increase in leaf chlorophyll content. The prior observation shows that, under non‐severe conditions, some of the genotypes assessed maintain their greenness until growth conditions return to normal, allowing plants to remobilize nutritional reserve storage in grains (Table 4). These observations confirm the findings of Abid et al. (2018). They also said that when plants were drought‐stressed, the amount of photosynthetic material in their leaves increased, and they quickly recovered after being watered again, which meant that production dropped less in the tolerant cultivar than in the susceptible ones. These findings, according to them, indicate that the plant's capacity to sustain functions during drought and recover quickly after re‐watering throughout vegetative stages is significant in determining wheat final performance. However, our results differed from those of Abid et al. (2018), who reported that severe drought stress treatments dramatically reduced thousand grain weight and grain yield per pot.

According to the clusters analysis (Figure 4), the genotypes tested displayed good adaptability to the specific conditions of the two locations (Ndiaye and Fanaye). Each of the four groups identified after the clusters analysis demonstrated this effectiveness, reflecting the parents' behavior in developing the current progenies. These clusters consisted of genotypes with high yield potential that had acclimated to the agroecological conditions of one or both of the rice‐growing locations. The remaining two groups were built based on particular agronomic traits recorded (Table 4). This result demonstrated rigor in the selection of lines and identification of the parents used. We selected the parents after serial screening in various environments over several years, incorporating information from seed specialists. Most of the time, researchers develop hybrid drought resistance by selecting both parental lines with higher yield potential, high combining ability, and drought tolerance contributing traits (Rauf et al. 2016; Mwadzingeni et al. 2017; Villa et al. 2011). After conducting crosses in the crossing block and observing the seed‐growth period in the pots up to F_3_ and subsequent families, they are evaluated in the hotspot related to their selection objectives. During the Stress Tolerant Rice for Africa and South Asia (STRASA) project conducted by AfricaRice, tolerant advanced lines with good yields were selected after Multilocation Environment Trials in the Breeding Taskforce (BTF). Countries where these lines are performed should eventually adopt, release, and register them to the national rice catalog. Rice self‐sufficiency in developing countries involves the development of superior high‐yielding tolerant to some stresses and agronomically suitable. The best genotypes in this study came from lines that were developed and showed the heterosis effect because of multiple genetic combinations reported in Table S1, Annex. The results of this study demonstrated the need to provide rice farmers with suitable varieties and agricultural best practices that can help them optimize their profitability. According to FAO (2022), Africa's average yield of rice is 2.4 t/ha, 33% lower than that of farmers in Southeast Asia (4.3 t/ha). Yuan et al. (2021) and Dossou et al. (2020) attributed such gaps to many aspects of agronomic management in African rice systems, such as insufficient fertilizer availability, insect, weed, and disease control challenges, and inefficient soil and water management. Yuan et al. (2024) suggested that the plan for raising rice output should be adjusted to each country's specificity. Those previous authors added that Africa will require a combination of yield intensification, similar to what occurred during Asia's Green Revolution, and modest area extension to enhance South Saharan Africa and avoid further rises in rice imports and costs.

## 5 Conclusion

This study highlights significant differences between the Fanaye and Ndiaye regions in key ecological and agronomic traits affecting rice farming in the Senegal River Delta. These differences emphasize the importance of considering local conditions and agricultural practices when planning to develop a breeding program. In terms of agroecological factors, Fanaye's conditions favor rice growth, resulting in more tillers and taller plants compared to Ndiaye. Additionally, rice plants in Fanaye exhibited a higher harvest index, indicating more efficient biomass production. Conversely, Ndiaye showed advantages in leaf chlorophyll content at 50% heading stage and thousand grain weight, suggesting grains well filled and heavy panicles. Economically, the findings have important implications for agricultural development. While grain yields were higher in Fanaye, the same genotypes in Ndiaye had a slightly lower harvest index, indicating less efficient use of agricultural resources. The reduced number of tillers, plant height, and grain yield in Ndiaye could lead to economic losses for farmers.

The study underscores the need to tailor agricultural practices to the specific conditions of each region. Targeted interventions to improve water management, soil fertility, and crop protection could enhance yields and sustain rice production. Additionally, research and development efforts focused on selecting and promoting rice varieties suited to each region could improve resilience to climate change and environmental stresses. Moreover, while the results emphasize interesting insights into the varietal performance, the most efficient and adapted genotypes can be considered for subsequent genetic and varietal evaluations, including mainly the analysis of their stability. This can lead to the consideration of several environmental conditions in time (i.e., different cropping seasons, years) and in space (i.e., various physical and biological environments, under abiotic stresses such as drought or salinity, as well as biotic stresses such as pests and diseases).

Meanwhile, the lines WAC18‐WAT15‐3‐1, WAC18‐WAT65‐1‐1, WAC13‐WAT32‐2‐1, and WAB2150‐TGR1‐WAT3‐1 produced the highest yields in Ndiaye, with 4752, 5589, 5589, 5644, and 6943 kg/ha, respectively. For Fanaye, the best genotypes were IR09N523, CT18919‐4‐2‐2‐2SR‐1P, CT18494‐4‐4‐3‐3‐1SR, WAB2125‐WACB‐1‐TGR1‐WAT1‐1, and CT19541‐13‐3‐1‐2P‐3P, with yields of 8824, 8984, 9014, 9639, and 8496 kg/ha, respectively. The authors recommend releasing these lines in African fields or using them as donors in breeding programs.

## Conflicts of Interest

The authors declare no conflicts of interest.

## Supporting information


Data S1.

## Data Availability

Data are provided with this article and Supporting Information.
