# Supplementary material for: Field Evaluation of Advanced Rice Lines for Adaptability to Drought and Heat in the Senegal River Valley
Source: Plant Environ Interact. 2025 Feb 16;6(1):e70034. doi: 10.1002/pei3.70034 (PMC11830397; doi:10.1002/pei3.70034)
Supplement: Supplementary file 1 — Data S1. [file PEI3-6-e70034-s001.docx]

Annex: **Table S1.** List of genotypes used during the assessment.

| **Entry** | **Genotypes** | **Category** |
| --- | --- | --- |
| 1 | WAB 2066-FKR4-WAC1-TGR5 B | Advanced line |
| 2 | WAB 1436-20N-3-B-FKR2-WAC1 | Advanced line |
| 3 | WAB 2102 - WAC1 -2-TGR4-WAT B6 | Advanced line |
| 4 | WAB 2060-3-FKR1-WAC2-TGR4-B | Advanced line |
| 5 | WAB 2060-FKR4-WAC1-TGR5-B | Advanced line |
| 6 | WAS 183-B-6-2-4 | Advanced line |
| 7 | WAB 2066-20-FKR2-WAC1-1-WAS-B | Advanced line |
| 8 | WAB 2066-6-FKR3-WAC1-1-WAS-B | Advanced line |
| 9 | WAB 2066-6-FKR4-WAC1-TGR1-B-WAT-B11 | Advanced line |
| 10 | WAB 2066-6-FKR4-WAC1-TGR1-B-WAT-B5 | Advanced line |
| 11 | WAB 2076-WAC1-TGR1-B | Advanced line |
| 12 | WAB 2076-WAC2-TGR1-B | Advanced line |
| 13 | WAS 62 B-B-14-1 | Advanced line |
| 14 | WAB 2151-TGR-WAT-B5 | Advanced line |
| 15 | WAB 2098-WAC2-1-TGR2-WAT B2 | Advanced line |
| 16 | WAB 1486-1N-B-FKR2-WACB | Advanced line |
| 17 | WAB 2098-WAC3-1-TGR1-WAT B3 | Advanced line |
| 18 | WAB 2098-WAC3-1-TGR2-WAT B1 | Advanced line |
| 19 | WAB 2099-WAC2-1-TGR2-WAT B5 | Advanced line |
| 20 | WAB 2099-WAC2-1-TGR2-WATB3 | Advanced line |
| 21 | WAB 2133-WACB-2-R1 WAS B | Advanced line |
| 22 | WAB 2102-WAC1-2-TGR5-WAT B6 | Advanced line |
| 23 | WAB 2102-WAC3-1-TGR1-WAT B2 | Advanced line |
| 24 | WAB 2102-WAC3-1-TGR1-WAT B7 | Advanced line |
| 25 | WAB 2057- 2-FKR4-WACB | Advanced line |
| 26 | WAB 2098-WAC3-1-TGR2-WATB6 | Advanced line |
| 27 | CT 16658-5-2-2SR-2-3-6MP | Advanced line |
| 28 | CT 18491-7-3-1-4-4P | Advanced line |
| 29 | WAB 2066-6-FKR4-WAC1-TGR1-B-WAT-B12 | Advanced line |
| 30 | CT 18494-4-4-3-3-1SR | Advanced line |
| 31 | CT 18506-11-9-3-3-2P | Advanced line |
| 32 | CT 18838-1-1-2-1SR-2P | Advanced line |
| 33 | CT 18919-4-2-2-2SR-1P | Advanced line |
| 34 | CT 19298(100)-1-2-3-1-1MP | Advanced line |
| 35 | CT 19298(100)-1-2-3-1-2MP | Advanced line |
| 36 | CT 19298(27)-1-11-1-2-3MP | Advanced line |
| 37 | CT 19298(83)-3-19-4-4-1MP | Advanced line |
| 38 | CT 19541-13-3-1-2P-3P | Advanced line |
| 39 | CT 19558-2-17-4P-3-1-1-M | Advanced line |
| 40 | CT 19561-3-1-1P-1-2-3-M | Advanced line |
| 41 | CT 19561-3-1-1P-2-1-1-M | Advanced line |
| 42 | CT 19561-3-1-1P-2-4-3-M | Advanced line |
| 43 | CT 21407-9P-5P-4SR-1 | Advanced line |
| 44 | DKA 21 | Variety |
| 45 | DKA 22 | Variety |
| 46 | DKA-M1 | Advanced line |
| 47 | FAROX 521-101-H1 | Advanced line |
| 48 | FAROX 521-119-H1 | Advanced line |
| 49 | FAROX 521-139-H1 | Advanced line |
| 50 | FAROX 521-146-H1 | Advanced line |
| 51 | FAROX 521-155-H1 | Advanced line |
| 52 | FAROX 521-156-H1 | Advanced line |
| 53 | Moroberekan | Variety |
| 54 | FAROX 521-288-H1 | Advanced line |
| 55 | FAROX 521-356-H1 | Advanced line |
| 56 | FAROX 521-357-H1 | Advanced line |
| 57 | FAROX 521-366-H1 | Advanced line |
| 58 | FAROX 521-384-H1 | Advanced line |
| 59 | WAB 1468 -14N-B-FKR5-FKRB | Advanced line |
| 60 | FAROX 521-82-H1 | Advanced line |
| 61 | FAROX 521-83-H1 | Advanced line |
| 62 | IR 06A150 | Advanced line |
| 63 | IR 08L222 | Advanced line |
| 64 | IR 09L336 | Advanced line |
| 65 | IR 09L348 | Advanced line |
| 66 | IR 09N523 | Advanced line |
| 67 | IR 67039-115-1-2 | Advanced line |
| 68 | IR 68 | Variety |
| 69 | IR 75395-2B-B19-RI-2-3-1-5-3 | Advanced line |
| 70 | IR 84649-21-15-1-B | Advanced line |
| 71 | IR 84649-275-3-2-B | Advanced line |
| 72 | IR 84649-81-4-B-B | Advanced line |
| 73 | IR83460-B-AJY4-2-SDO3 | Advanced line |
| 74 | IRRI 123 | Variety |
| 75 | WAB 1529-7-B-B-FKR4-WAC1-2-TGR2-WAT7-1 | Advanced line |
| 76 | WAB 1529-7-B-B-FKR4-WAC1-2-TGR3-WAT1-1 | Advanced line |
| 77 | WAB 1572-10-B-B-FKR4-WAC1-1-TGR2-WAT10-1 | Advanced line |
| 78 | WAB 1572-10-B-B-FKR4-WAC1-1-TGR4-WAT14-1 | Advanced line |
| 79 | WAB 1573-22-B-B-FKR4-2-WAC1-TGR3-WAT9-1 | Advanced line |
| 80 | WAB 2061-2-FKR1-WAC2-TGR4-B | Advanced line |
| 81 | WAB 2066-14-FKR3-RI-WAC1-WASB | Advanced line |
| 82 | WAB 2066-2-FKR-WACB | Advanced line |
| 83 | WAB 2075-WAC5-FKR2-3-TGR1 | Advanced line |
| 84 | WAB 2081-WAC2-2-TGR2-WAT1-8 | Advanced line |
| 85 | WAB 2081-WAC2-2-TGR2-WAT1-9 | Advanced line |
| 86 | WAB 2098-WAC1-FKR2-4-TGR1 | Advanced line |
| 87 | WAB 2098-WAC3-1-TGR1-WAT3-10 | Advanced line |
| 88 | WAB 2098-WAC3-1-TGR1-WAT3-8 | Advanced line |
| 89 | WAB 2099-WAC1-FKR1-1-TGR1 | Advanced line |
| 90 | WAB 2099-WAC1-FKR2-1-TGR1 | Advanced line |
| 91 | WAB 2099-WAC1-FKR2-4-TGR1 | Advanced line |
| 92 | WAB 2099-WAC1-1-TGR1-WAT2-7 | Advanced line |
| 93 | WAB 2125-WAC B-1-TGR1-WAT1-1 | Advanced line |
| 94 | WAB 2135-WAC B-2-TGR2-WAT1-1 | Advanced line |
| 95 | WAB 2135-WAC B-2-TGR2-WAT3-1 | Advanced line |
| 96 | WAB 2150-TGR1-WAT3-1 | Advanced line |
| 97 | WAB 2152-TGR2 | Advanced line |
| 98 | WAB 2152-TGR4 | Advanced line |
| 99 | WAB 2153-TGR2 | Advanced line |
| 100 | WAB 2156-TGR1-WAT2-1 | Advanced line |
| 101 | WAC 11-TGR6 | Advanced line |
| 102 | WAC 12-TGR2 | Advanced line |
| 103 | WAC 13-TGR4 | Advanced line |
| 104 | WAC 13-TGR5 | Advanced line |
| 105 | WAC 13-WAT21-2-1 | Advanced line |
| 106 | WAC 13-WAT32-2-1 | Advanced line |
| 107 | WAC 18-WAT15-3-1 | Advanced line |
| 108 | WAC 18-WAT65-1-1 | Advanced line |
| 109 | WAC 29-TGR1 | Advanced line |
| 110 | IR 50 | Variety |
| 111 | WAC12-TGR2 | Advanced line |
| 112 | WAB 2128-WAC B-1-TGR1-WAT B2 | Advanced line |
| 113 | WAB 1522-5-B-B-FKR3-WAC1-1-WAS-B | Advanced line |
| 114 | WAB 2056-8-FKR4-WAC B | Advanced line |
| 115 | WAC11-TGR6 | Advanced line |
| 116 | WAC29-TGR1 | Advanced line |
| 117 | WAB 1572-10-B-B-FKR4-WAC1-1-TGR2-WAT10-1 | Advanced line |
| 118 | WAB2152-TGR2 | Advanced line |
| 119 | Sahel 108 | Variety |
| 120 | NERICA-S-36 | Variety |
